# Impact of tooth loss on walking speed decline over time in older adults: a population-based cohort study

**DOI:** 10.1007/s40520-016-0630-6

**Published:** 2016-09-28

**Authors:** Anna-Karin Welmer, Debora Rizzuto, Marti G. Parker, Weili Xu

**Affiliations:** 1 Department of Neurobiology, Care Sciences and Society, Aging Research Center, Karolinska Institutet and Stockholm University, Gävlegatan 16, 9tr, SE113-30 Stockholm, Sweden; 2 Karolinska University Hospital, Stockholm, Sweden; 3 Department of Epidemiology and Biostatistics, Tianjin Medical University, Tianjin, China

**Keywords:** Tooth loss, Aging, Walking speed, Chronic disease, Swedish National Study on Aging and Care in Kungsholmen (SNAC-K)

## Abstract

**Background:**

Tooth loss has been linked to poor health such as chronic diseases and mobility limitations. Prospective evidence on the association between tooth loss and walking speed decline is however lacking.

**Aims:**

To examine the impact of tooth loss on walking speed over time and explore whether inflammation may account for this association.

**Methods:**

This study included 2695 persons aged 60 years and older, who were free from severe mobility limitation at baseline. Information on dental status was assessed through self-report during the nurse interview at baseline. Walking speed baseline and at 3- and 6-year follow-ups was assessed when participants walked at their usual pace. Covariates included age, sex, education, lifestyle-related factors, and chronic diseases. Blood samples were taken, and C-reactive protein (CRP) was tested.

**Results:**

At baseline, 389 (13.1 %) participants had partial tooth loss and 204 (6.9 %) had complete tooth loss. Mixed-effects models showed that tooth loss was associated with a greater decline in walking speed over time after adjustment for lifestyle-related factors and chronic diseases (*p* = 0.001 for interaction between time and tooth loss on walking speed decline); however, when further adjusting for inflammation (CRP), the association was attenuated and no longer significant.

**Conclusion:**

Tooth loss was associated with an accelerated decline in walking speed in older adults. Inflammation may play a role in the association between tooth loss and walking speed decline.

## Introduction

Tooth loss in older adults is mainly caused by periodontal disease [1–3] and may reflect oral health over the life course. It can be both a symptom and a cause of further health complications, such as cardiovascular disease (CVD) [4], cognitive impairment [5], and disability [6, 7] in older adults. Thus, it has been suggested that tooth loss may be an indicator of accelerated aging [6].

Periodontal disease and tooth loss have been linked to impaired physical functions such as poor muscle strength, balance and walking speed, and the presence of frailty in cross-sectional studies [8–11]. Walking speed is a clinically useful measure in the care of older adults. It is a valid marker of mobility and overall health status and an independent predictor of adverse outcomes such as disability, dementia, and mortality [12–14]. Impaired walking speed is likely to be a consequence of different exposures occurring throughout the life course. So far, few studies have examined longitudinal associations between oral health and physical function. Prospective studies have shown that periodontitis and tooth loss are associated with decline in physical functions such as balance and muscle strength [15, 16], and incident disability [6].

A possible pathway for this association may be long-standing infection and inflammation associated with periodontal disease [17], which can increase the risk of sarcopenia and physical impairment [18, 19]. Moreover, periodontal disease is a risk factor for chronic diseases, such as CVD, which is strongly associated with impaired physical function [4, 20]. However, questions remain about the association of tooth loss with walking speed changes and the role of inflammation in such an association. In the present study, we aimed to examine the impact of tooth loss on walking speed over time and to explore whether inflammation may account for this association, adjusting for chronic diseases, and other potential confounders.

## Methods

### Study population

Data were gathered from the population-based Swedish National Study on Aging and Care in Kungsholmen (SNAC-K) [21]. The SNAC-K study population consists of persons aged 60 years and older living at home or in an institution in the Kungsholmen district of central Stockholm. The sample was randomly selected from 11 different age groups: 60, 66, 72, 78, 81, 84, 87, 90, 93, 96, and 99 or older. Follow-up is performed every 6 years for younger cohorts (60–78 years) and every 3 years for older cohorts (age ≥78 years). The baseline data collection was conducted 2001 through 2004, the first follow-up for the older cohorts was conducted 2004 through 2006 (3-year follow-up), and the second follow-up for the older cohorts and the first follow-up for the younger cohorts were completed in 2010 (6-year follow-up).

Of the 5111 persons who were initially selected to be invited for participation, 521 were not eligible (200 died before start of the study, 262 had no contact information, 32 had moved, 23 did not speak Swedish, and 4 were deaf). Among the remaining 4590 persons, 3363 (73.3 %) participated at the baseline examination. We excluded persons with missing values in walking speed (*n* = 258), who were unable to walk at baseline (*n* = 145), or with missing values in tooth loss (*n* = 265), thus the analytical sample included 2695 participants at baseline (1586 aged <78 years, and 1109 aged ≥78 years). The analytical sample was significantly younger (baseline mean age ± SD 73.1 ± 10.5 vs. 79.4 ± 11.5, *p* < 0.001) than the excluded participants, included fewer women (62.7 % vs. 70.9 %, *p* < 0.001), and was better educated (for university education 35.6 % vs. 22.7 %, *p* < 0.001).

**Fig. 1:**
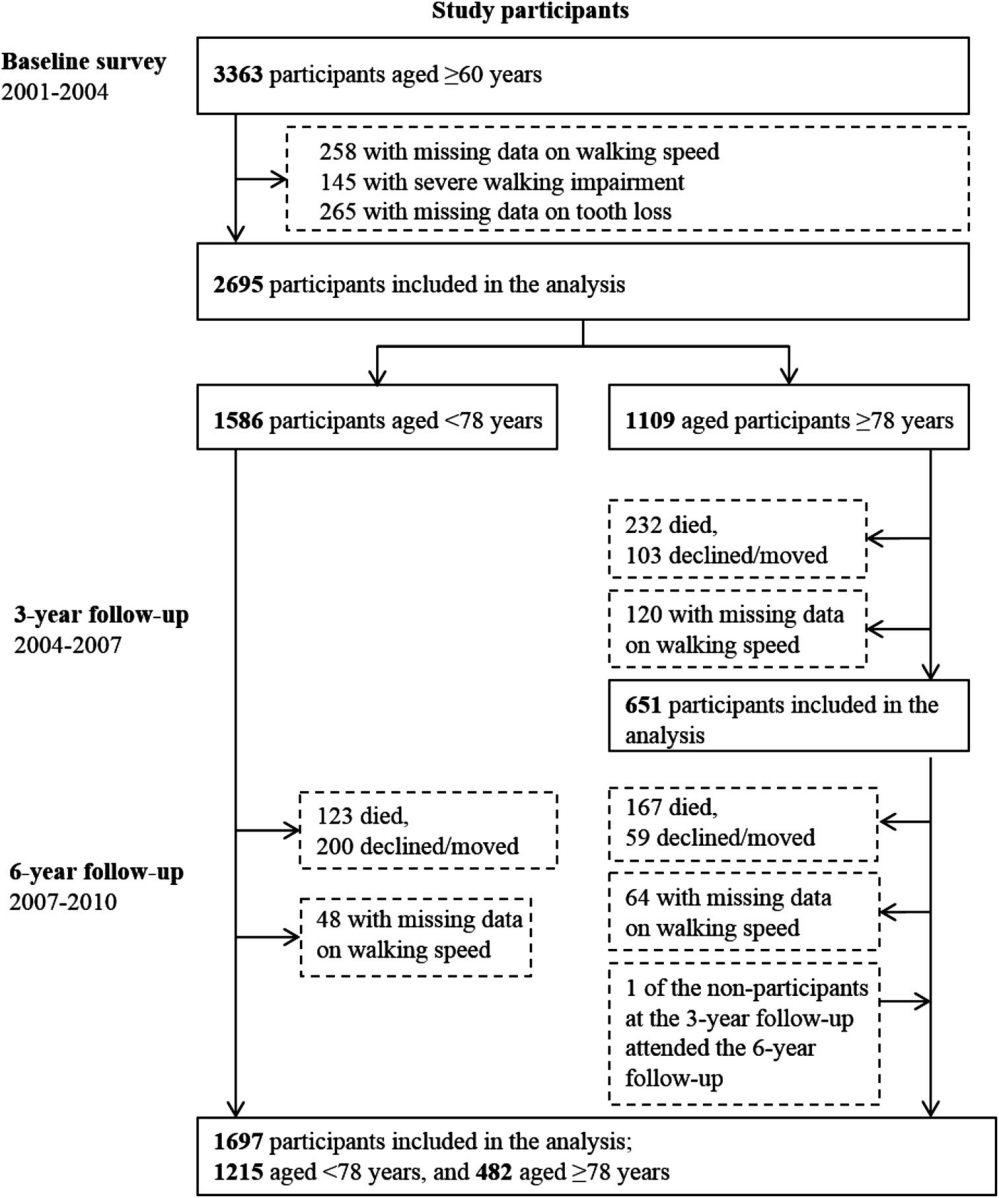
Flowchart of the study population in the Swedish National Study on Aging and Care in Kungsholmen, Stockholm, Sweden

In the older cohorts, 771 participated at the 3-year follow-up; of those 120 had missing data on walking speed, thus the analytical sample consisted of 651 participants at the 3-year follow-up. In the younger cohorts, 1263 participated at the 6-year follow-up; of those 48 had missing data on walking speed. In the older cohorts, 546 participated at the 6-year follow-up; of those 64 had missing data on walking speed. Thus, the analytical sample consisted of 1697 participants (1215 aged <78 years, and 482 aged ≥78 years) at the 6-year follow-up (Fig. 1).

### Ethics

The study was approved by the Regional Ethical Review Board in Stockholm, Sweden. Informed consent was obtained from all individual participants included in the study. If the participant was cognitively impaired, a proxy (usually a close family member) was asked for consent, in addition to asking the participant.

### Data collection

Data included information on dental status, walking speed, demographic factors, and lifestyle, collected by nurses; and information on medical conditions assessed by physicians through clinical examination, self-reported medical history, and laboratory data. The International Classification of Diseases-10th Revision (ICD-10) was used to classify diseases. Walking speed and C-reactive protein (CRP) were assessed at baseline and at the 3- and 6-year follow-ups. Tooth loss status and covariates were all collected at baseline.

*Walking speed assessment* Participants were asked to walk 6 m in their usual pace, or 2.4 m if the participant reported walking quite slowly. A walking aid was allowed. For the analyses, the walking speed reflects the time from whichever length walked, presented in meters per second. When participants were unable to walk without personal support, a value of 0 m/s was recorded.

*Tooth loss* was examined through the following question: ‘Do you have your own natural teeth or removable denture?’ The participants were categorized in three groups as follows: (1) no tooth loss: having only own teeth; (2) partial tooth loss: having own teeth with removable denture, or implants; and (3) complete tooth loss with or without full dentures in both jaws [21].

Blood samples were taken from all participants, and CRP in plasma was tested following a standard procedure. *High CRP* was defined as CRP levels higher than 5 mg/L [22]. CRP data were available for 2556 (94.8 %) of the 2695 participants at baseline, for 613 (94.2 %) of the 651 participants at the 3-year follow-up, and for 1626 (95.8 %) of the 1697 participants at the 6-year follow-up.

*Chronic diseases CVD* included coronary heart disease, atrial fibrillation, and heart failure and was ascertained based on information from clinical examination, electrocardiogram, and medical history. *Stroke* and *cancer* were defined based on medical history. *Hypertension* was defined as blood pressure ≥140/90 mmHg or current use of antihypertensive agents [Anatomical Therapeutic Chemical (ATC) codes C02, C03, C07, C08, and C09] [23]. *Dementia* was diagnosed according to the Diagnostic and Statistical Manual of Mental Disorders (DSM)-IV criteria [24]. *Diabetes* was ascertained based on medical history, clinical examination, and current use of oral glucose-lowering agents or insulin injection (ATC code A10), or having a glycosylated hemoglobin level ≥5.4 % [25]. *Musculoskeletal disease* included arthrosis and arthritis and was defined based on medical history.

*Demographic factors* included data on age, gender, and education. Education was defined as the highest level of formal education and was categorized into elementary school, high school, or university. *Lifestyle*-*related factors* included body mass index (BMI), smoking status, alcohol consumption, and physical exercise. Height and weight were measured by nurses. *BMI* was calculated by dividing weight in kilograms by height in meters squared and was categorized as underweight (<20), normal (20–24.9, reference), overweight (25–29.9), and obesity (≥30 kg/m^2^) [26]. *Smoking status* was categorized into never, former, and current smoking. *Alcohol consumption* was categorized into no or occasional, light-to-moderate (1–14 drinks per week for men or 1–7 drinks per week for women) or heavy (≥15 drinks per week for men or ≥8 drinks per week for women). *Physical exercise* was measured by two survey questions: (1) ‘How often did you exercise with light intensity (e.g., walks, short bike rides, light gymnastics, golf) in the last 12 months?’ and (2) ‘How often did you exercise more intensively (e.g., brisk walking, jogging, heavy gardening, long bike rides, intense gymnastics, skiing, swimming) in the last 12 months?’. Based on research recommendations, the participants were categorized in three groups according to the levels of the activities: (1) inadequate: ≤2–3 times per month in light and/or intensive activity; (2) health enhancing: light exercise several times per week or every day; and (3) fitness enhancing: moderate/intense exercise several times per week or every day [27].

### Data analysis

Baseline characteristics of the participants by tooth loss status were compared using one-way ANOVA for continuous variables and chi-square for categorical variables. The association between tooth loss and change in walking speed across all three testing occasions (baseline, 3 and 6 years) was analyzed using linear mixed-effects models. The within-person residual covariance matrix was evaluated with the unstructured correlation structure. Each model included age, sex, education, and the follow-up time as an indicator variable, expressed as the three testing occasions. We first examined the average change in walking speed over the study period in the total sample. Secondly, we investigated the average change in walking speed over the study period by baseline tooth loss status. We included the interaction between tooth loss and follow-up time on change in walking speed. Interactions were tested by including simultaneously the independent variables and their cross-product variables in the same model. Mixed-effect models were performed with stepwise adjustments as following: (1) adjustment for age, sex, education, and follow-up time; (2) additional adjustments for baseline lifestyle-related factors (BMI, smoking, alcohol consumption, and physical exercise) and chronic diseases (CVD, stroke, hypertension, diabetes, musculoskeletal disease, dementia, and cancer); and (3) further additional adjustments for baseline and follow-up levels of CRP. Statistical analyses were performed with Stata, version 14.0 (StataCorp, TX, USA).

## Results

Of the 2965 participants, 389 (13.1 %) had partial tooth loss, and 204 (6.9 %) had complete tooth loss at the baseline examination. Participants with partial or complete tooth loss were more likely to be older, female, and less educated, and have less alcohol consumption, slower walking speed, less physical activity, more often CVD, hypertension, diabetes, and dementia, and high CRP compared to participants without tooth loss (Table 1).

**Table 1:** Characteristics of the study participants by tooth loss status at baseline (*n* = 2695)

Characteristics	Tooth loss status at baseline						*p* value
	No (*n* = 2102)		Partial (*n* = 389)		Complete (*n* = 204)		
	*n*	Mean ± SD or %	*n*	Mean ± SD or %	*n*	Mean ± SD or %	
Age	2102	70.9 ± 9.7	389	79.3 ± 9.5	204	84.9 ± 8.4	<0.001
Women	1287	61.2	259	66.6	143	70.1	0.010
Education							
Elementary school	212	10.1	118	30.3	89	43.8	
High school	1024	48.7	199	51.2	92	45.3	
University	866	41.2	72	18.5	22	10.8	<0.001
Walking speed (m/s)	2102	1.1 ± 0.4	389	0.86 ± 0.4	204	0.67 ± 0.3	<0.001
Body mass index (kg/m2)							
<20 (underweight)	93	4.6	30	8.3	22	12.9	
20–24.9 (normal weight)	841	41.1	129	35.5	72	42.1	
25–29.9 (overweight)	844	41.3	161	44.4	52	30.4	
≥30 (obesity)	266	13.0	43	11.9	25	14.6	<0.001
Smoking status							
Never	977	46.9	170	43.8	92	45.3	
Former	823	39.5	152	39.2	73	36.0	
Current	285	13.7	66	17.0	38	18.7	0.169
Alcohol consumption							
No	568	27.2	187	48.2	140	69.0	
Moderate	1129	54.1	147	37.9	54	26.6	
Heavy	391	18.7	54	13.9	9	4.4	<0.001
Physical exercise							
Inactive	498	23.7	153	39.3	103	50.5	
Health enhancing	1086	51.7	189	48.6	84	41.2	
Fitness enhancing	518	24.6	47	12.1	17	8.3	<0.001
CVD	532	25.3	165	42.4	122	59.8	<0.001
Stroke	82	3.9	22	5.7	13	6.4	0.099
Hypertension	1505	71.9	318	81.8	175	85.8	<0.001
Diabetes	177	8.4	51	13.1	26	12.8	0.004
Musculoskeletal disease	277	13.2	58	14.9	29	14.2	0.643
Dementia	53	2.5	21	5.4	31	15.2	<0.001
Cancer	144	6.9	29	7.5	15	7.4	0.898
High C-reactive protein	351	17.5	84	23.4	51	27.6	<0.001

*CVD* cerebrovascular disease (coronary heart disease, atrial fibrillation, and heart failure)

Linear mixed-effects models revealed significant interactions between tooth loss status and follow-up time in association with walking speed, such that tooth loss was associated with a greater decline in walking speed (Table 2). The gradient difference in walking speed decline by tooth loss status persisted even after adjusting for lifestyle-related factors and chronic diseases; however, when further adjusting for CRP, the association between tooth loss and walking speed decline was attenuated and no longer significant (Table 2). At baseline, we found significant differences in walking speed between people with no tooth loss and people with partial tooth loss (*p* = 0.015), and between people with no tooth loss and people with complete tooth loss (*p* < 0.001), when adjusting for demographic factors (not shown). In the multi-adjusted models, these differences were attenuated and no longer statistically significant. In additional analysis, we further adjusted for (1) self-reported chewing problems (*n* = 2690) and 2) nutritional status (using the Mini Nutritional Assessment-Short Form [28], *n* = 2578), similar results were obtained to those from initial analyses (not shown). Furthermore, we performed gender-stratified analyses, and the results were similar for men and women.

**Table 2:** Change in walking speed over time, in the total sample, and by tooth loss status

	Change in walking speed (m/s)		
	Adjusted for demographic factors	Adjusted for demographic and lifestyle-related factors, and chronic diseases	Adjusted for demographic and lifestyle-related factors, chronic diseases, and CRP^a^
	β-Coefficient (95 % CI)	β-Coefficient (95 % CI)	β-Coefficient (95 % CI)
Total sample			
Intercept (baseline speed)	1.04 (1.00–1.08)	1.11 (1.06–1.16)	1.12 (0.99–1.25)
Average change over 3 years^b^	−0.06 (−0.08 to −0.04)	−0.06 (−0.08 to −0.04)	−0.10 (−0.13 to −0.08)
Average change over 6 years	−0.15 (−0.16 to −0.13)	−0.15 (−0.16 to −0.14)	−0.27 (−0.29 to −0.24)
By tooth loss status			
*p* value for interaction between time and tooth loss	0.003	0.001	0.603
Among people with no tooth loss at baseline (*n* = 2102)			
Intercept (baseline speed)	1.07 (1.02–1.12)	1.12 (1.06–1.17)	1.14 (1.00–1.28)
Average change over 3 years^b^	−0.05 (−0.08 to −0.03)	−0.05 (−0.08 to −0.03)	−0.10 (−0.13 to −0.06)
Average change over 6 years	−0.14 (−0.15 to −0.12)	−0.14 (−0.15 to −0.12)	−0.27 (−0.30 to −0.24)
Among people with partial tooth loss at baseline (*n* = 389)			
Intercept (baseline speed)	1.03 (0.97–1.08)	1.10 (1.05–1.16)	1.11 (0.96–1.25)
Average change over 3 years^b^	−0.06 (−0.11 to −0.02)	−0.07 (−0.11 to −0.02)	−0.10 (−0.16 to −0.05)
Average change over 6 years	−0.18 (−0.22 to −0.14)	−0.20 (−0.24 to −0.15)	−0.25 (−0.31 to −0.20)
Among people with complete tooth loss at baseline (*n* = 204)			
Intercept (baseline speed)	0.97 (0.91–1.03)	1.08 (1.01–1.15)	1.12 (0.96–1.29)
Average change over 3 years^b^	−0.13 (−0.19 to −0.07)	−0.11 (−0.18 to −0.05)	−0.12 (−0.21 to −0.03)
Average change over 6 years	−0.25 (−0.32 to −0.18)	−0.26 (−0.33 to −0.19)	−0.34 (−0.43 to −0.24)

Demographic factors included age, sex, and education

Lifestyle-related factors included body mass index, smoking, alcohol consumption, and physical exercise

Chronic diseases included cardiovascular disease, stroke, hypertension, diabetes, musculoskeletal disease, dementia, and cancer

*CI* confidence interval, *CRP* C-reactive protein

^a^Adjusted for CRP at baseline and follow-ups

^b^Includes only participants aged ≥78 years at baseline

Finally, sensitivity analysis was performed in which participants with stroke or dementia were excluded from the analytical sample (*n* = 211), and similar results were obtained to those from initial analyses.

## Discussion

In this large prospective cohort study of people aged ≥60 years, we found that people with tooth loss showed a greater decline in walking speed over time, independent of lifestyle and chronic diseases, but not independent of CRP, suggesting that inflammation may play a role in the association between tooth loss and walking speed decline.

To the best of our knowledge, this is the first study that examined the effect of tooth loss on walking speed over time. The results support previous research that links poor dentition to physical impairments [6, 8–10, 15, 16]. Tooth loss may be an indicator of low physiological reserve [29] resulting from cumulative effects of unfavorable exposures throughout the life course, which also has adverse effects on walking speed. Common risk factors for periodontitis and walking speed decline are, for example, lifestyle-related factors such as smoking and obesity [30]. Moreover, periodontitis is a risk factor for chronic disease, such as CVD, which is strongly associated with impaired walking speed [4, 20]. In the present study, the association between tooth loss and walking speed decline persisted when adjusting for lifestyle and chronic diseases, which diminishes the plausibility of lifestyle and chronic disease as possible pathways. However, we did not have information on the length or severity of diseases. Moreover, we cannot exclude changes in covariates such as an increasing number or severity of diseases over time in people with tooth loss as a possible mechanism.

Since periodontal disease is the major cause of tooth loss in older adults [1–3], tooth loss may be considered to be an indirect marker of previous and current periodontal disease. Our results support the hypothesis that long-standing inflammation associated with periodontal disease may be a possible link between dental status and physical impairment [15]. Long-standing inflammation may cause physical impairment through sarcopenia and systemic damage such as atherosclerosis [2, 19]. Subclinical atherosclerosis may play an important role in motor function, thus proving one plausible pathway to the link between tooth loss and declining walking speed, even in the absence of chronic diseases [31]. It must, however, be pointed out that tooth loss is a crude measure of oral health since it may be influenced by other factors than periodontitis, such as dental extraction policies. We did not have information on periodontal disease, which limit our possibilities of examining the underlying mechanisms of the reported associations. Furthermore, although the gradient difference in walking speed decline by tooth loss status became non-significant when adjusting for CRP, the differences in point estimates for the groups did not differ much between the model adjusted for CRP and the model without adjustment for CRP. Thus, no ultimate conclusion can be drawn on the role of CRP in explaining this association.

Although our results indicate that tooth loss is predictive of declining walking speed, a previous study found that self-reported tiredness or need of help in mobility increased the likelihood of not using dental services [7]. Thus, it is possible that the association between oral health and mobility is bidirectional. Indeed, it has been suggested that there may be a common parallel aging of oral health and other functional capacities, such that oral impairments and functional limitations, are interrelated [32].

Strength of this study includes the longitudinal design and the large population-based sample of older adults, living at home or in an institution. Moreover, walking speed was objectively tested and we used different sources of medical diagnoses, including direct clinical examination, thus limiting potential biases. However, there are also some limitations to this study. One concern that tooth loss was collected through self-report. Although previous studies support the validity of self-reported oral health in older adults [33], the assessment method may have led to an underestimation of the true associations between tooth loss and walking speed. The sample consisted of relatively highly educated older adults, without severe mobility limitations at baseline. This may limit the generalization of our results to quite high functioning cohorts of older adults, living in relatively affluent urban areas. Finally, a previous Swedish study examining cohort effects of edentulousness found that the rates of edentulousness became increasingly lower for successively later cohorts [34]. Thus, the associations reported in the present study may not be generalizable to future cohorts of older adults. Despite the limitations, this study adds to our understanding of the multifactorial causes of declining mobility in old age.

In conclusion, our results indicate that tooth loss is an early marker of a greater decline in walking speed over time in older adults and that inflammation may play a role in the association between tooth loss and walking speed decline. This study adds to our understanding of the multifactorial causes of declining mobility in old age.
